# Complementary or Alternative Medicine as Possible Determinant of Decreased Persistence to Aromatase Inhibitor Therapy among Older Women with Non-Metastatic Breast Cancer

**DOI:** 10.1371/journal.pone.0081677

**Published:** 2013-12-18

**Authors:** Laetitia Huiart, Anne-Deborah Bouhnik, Dominique Rey, Frédérique Rousseau, Frédérique Retornaz, Mégane Meresse, Marc Karim Bendiane, Patrice Viens, Roch Giorgi

**Affiliations:** 1 Unité de Soutien Méthodologique, CHU La Réunion, Saint-Denis, France; 2 U912 (SESSTIM), INSERM, Marseille, France; 3 Université Aix Marseille, IRD, UMR-S912, Marseille, France; 4 ORS PACA, Observatoire Régional de la Santé Provence Alpes Côte d'Azur, Marseille, France; 5 Medical Oncology Department, Institut Paoli-Calmettes, Marseille, France; 6 Centre Gérontologique Départemental, Marseille, France; 7 Université Aix Marseille, Centre de Recherche en Cancérologie de Marseille, INSERM U891, Marseille, France; 8 Service Biostatistique et Technologies de l'Information et de la Communication, Hôpital de la Timone, Assistance Publique – Hopitaux de Marseille, Marseille, France; Texas Tech Univ School of Pharmacy, United States of America

## Abstract

**Purpose:**

Aromatase inhibitor therapy (AI) significantly improves survival in breast cancer patients. Little is known about adherence and persistence to aromatase inhibitors and about the causes of treatment discontinuation among older women.

**Methods:**

We constituted a cohort of women over 65 receiving a first AI therapy for breast cancer between 2006 and 2008, and followed them until June 2011. Women were selected in the population-based French National Health Insurance databases, and data was collected on the basis of pharmacy refills, medical records and face-to-face interviews. Non-persistence to treatment was defined as the first treatment discontinuation lasting more than 3 consecutive months. Time to treatment discontinuation was studied using survival analysis techniques.

**Results:**

Overall among the 382 selected women, non-persistence to treatment went from 8.7% (95%CI: 6.2–12.1) at 1 year, to 15.6% (95%CI: 12.2–19.8) at 2 years, 20.8% (95%CI: 16.7–25.6) at 3 years, and 24.7% (95%CI: 19.5–31.0) at 4 years. In the multivariate analysis on a sub-sample of 233 women with available data, women using complementary or alternative medicine (CAM) (HR = 3.2; 95%CI: 1.5–6.9) or suffering from comorbidities (HR = 2.2; 95%CI: 1.0–4.8) were more likely to discontinue their treatment, whereas women with polypharmacy (HR = 0.4; 95%CI: 0.2–0.91) were less likely to discontinue. In addition, 13% of the women with positive hormonal receptor status did not fill any prescription for anti-hormonal therapy.

**Conclusion:**

AI therapy is discontinued prematurely in a substantial portion of older patients. Some patients may use CAM not as a complementary treatment, but as an alternative to conventional medicine. Improving patient-physician communication on the use of CAM may improve hormonal therapy adherence.

## Introduction

In the field of oncology, the use of oral therapy is on the rise, and treatment adherence is under increasing scrutiny [1], [2]. Oral adjuvant hormonal therapy in hormone-responsive early breast cancer (BC) reduces the risk of recurrence and increases survival rates [3]. Aromatase Inhibitors (AIs) were shown to improve disease-free survival as compared to tamoxifen in post-menopausal women [4], [5], [6], [7], [8]. They therefore constitute an alternative to adjuvant treatment of early BC [9], [10].

Non-adherence and early discontinuation of hormonal treatment are likely to affect treatment efficacy in BC patients [3], [11], [12], [13]. In a recently published meta-analysis on 29 observational studies, discontinuation rates for AIs ranged from 31 to 73% over the treatment period [14], [15]. These heterogeneous results are derived either from pharmacy databases or from samples of limited size using self-reported measures of adherence. Studies on the determinants of non-adherence are therefore limited either by self-reported measures of adherence - known to largely overestimate adherence - or by access to a restricted number of covariates in available pharmacy databases. While most database studies in pharmacoepidemiology use high quality pharmacy and medical data, they only rarely link these with medical records or with patient questionnaires. Combining data sources is necessary to improve our understanding of medication consumption patterns in conjunction with the patients' broader environment [16].

Our objective was to combine multiple sources of data to obtain a description of adherence and persistence with AI treatment (along with their determinants) in a population-based cohort of post-menopausal women with primary BC. Specifically, we evaluated adherence to treatment based on drug delivery records in pharmacy databases, and took into account determinants not available in such databases by collecting longitudinal psychosocial data directly from the patient.

## Methods

### Primary data source

The primary data source for patient selection was provided by The French National Health Insurance System (NHIS). The NHIS delivers universal health coverage; hence its database is population-based, i.e. it covers all segments of the population. Data was obtained from the NHIS which provides health insurance to 98% of the French population. The study area comprised 3 French administrative districts (Alpes-Maritimes, Bouches-du-Rhone, Var), which correspond to a population of approximately 4 million inhabitants.

In France, hormonal therapy treatment is available only in pharmacy by medical prescription. Level of reimbursement varies according to drug and patient characteristics. BC patients are reported to the NHIS by their physician and receive all treatment free of charge. BC patients were identified through this medical registry that includes all patients eligible for full treatment coverage. This database can be linked to the pharmacy refill database thanks to a unique identifier allocated to every adult individual.

Detailed description of the NHIS database is provided elsewhere [17].

### Study Population

The ELIPPSE 65 cohort was constituted in order to document the medium and long-term psychosocial impact of BC on women over 65. Eligible participants were women with a biopsy-proven diagnosis of primary BC who had been registered in the NHIS database between October 2006 and December 2008. Women were excluded if they had a previous history of BC; if they suffered from severe cognitive impairment, deafness or acute mental disorder; or if they were unable to answer a questionnaire. Follow-up was interrupted in June 2011.

We restricted the analysis to cohort members who received at least one supply of AI treatment for BC, as registered in the NHIS medication database.

### Ethics statement

All participants provided a written informed consent to participate in the study. The study was approved by the French National Committee on Information Technology and Individual Liberties (CNIL).

### Data Collection

#### Patient Data

In the month following BC diagnosis, all eligible women registered in the NHIS database were sent an explanatory letter about the survey by the NHIS medical advisory board and invited to return their written consent. Women who agreed to participate in the ELIPPSE 65 cohort were then sent a short self-administered questionnaire by mail, which included questions on patient characteristics and the circumstances of patient diagnosis. The follow-up comprised an in-home, face-to-face interview at 10 months. These interviews provided data on socio-demographic and psychosocial characteristics, drug use, complementary and alternative medicine (CAM) use, medical follow-up, and a geriatric assessment conducted as part of this study.

The geriatric assessment focused on independent living skills with the Activities of Daily Living scale (ADL) [18], the Instrumental Activities of Daily Living scale (IADL) [19], and cognitive functions by the Mini-Cog [20]. Disability in the ADL/IADL scales was defined as the need for assistance in carrying out at least one activity mentioned in the corresponding scale.

Women were classified as having depressive symptoms if their GDS-15 score was 5 or higher [21].

Polypharmacy was defined as receiving concurrently 4 types of medication or more, as reported in the patient questionnaire.

#### Medical Record

In parallel, a medical record was obtained from the patient's physician. A first medical questionnaire was sent to the physician who had established the diagnosis and/or who was in charge of cancer treatment. This questionnaire covered the patient's medical history, concurrent diseases, pathological assessment of BC and detailed information on cancer treatment. Subsequent medical questionnaires were sent to the patient's physician to obtain information on current treatment, BC recurrence, hospitalizations and vital status.

#### Pharmacy refill data

The NHIS pharmacy refill database was used to collect data on AI dispensed. All reimbursement claims are submitted to the NHIS at the moment of refill via a single electronic system. Drug characteristics, including name, dosage, and number of pills are recorded in the database. In France, AI are delivered free of charge for cancer patients. They are dispensed monthly even though a prescription may cover up to 3 months.

### Definition of Medication Adherence and Persistence Measures

These 2 outcomes were defined from the pharmacy refill database. Adherence was measured by the Medication Possession Ratio (MPR) [22], [23], which is defined as the number of days of treatment divided by the number of days between cohort entry and end of follow-up. For each woman, the number of days of treatment was calculated from the number of tablets dispensed combined with dosage instructions. Overlapping prescriptions were included in the calculation. Patients with an MPR below 80% were considered non-adherent [14], [24], [25], [26].

We defined non-persistence to treatment as the first treatment discontinuation lasting more than 3 consecutive months.

### Statistical Analysis

Descriptive statistics were computed for continuous data (mean, +/−standard deviation (sd) or median, 25–75% Inter Quartile Range (IQR)) and categorical data (sample size and percentage). Time to treatment discontinuation was calculated using Kaplan-Meier estimates to account for censored data. Women's follow-up was censored at the time of death, BC recurrence or contralateral BC, switching to a different anti-hormonal treatment, or on June 2011, whichever came first. Results were expressed as cumulative probabilities of treatment discontinuation with 95% confidence intervals (CI). Kaplan-Meier estimates of treatment discontinuation of different sub-groups were compared using the log-rank test.

Independent predictors of AI discontinuation were identified using a Cox proportional hazards model. The proportional hazards assumption was checked by examining the log-minus-log survival plot drawn up for each cofactor. We included in the initial multivariate model variables with a *p*-value<0.20 in the univariate analysis. We kept in the final model only variables that were still significantly associated with AI intake discontinuation with a *p*-value<0.10.

Statistical analyses were performed using the STATA version 9.0 software program (STATA Corp, College Station, TX).

## Results

**Figure 1:**
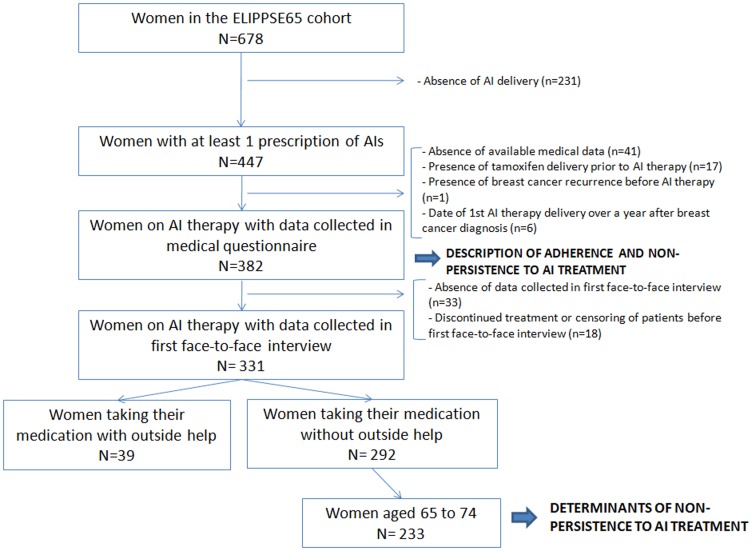
Study sample selection – ELIPPSE 65 cohort.

From October 2006 to December 2008, 678 women over 65 with BC were included in the ELIPPSE 65 cohort. A prescription of AIs was filled at least once by 447 of these women (65.9%). Among the latter, 65 (14.5%) were found to be non-eligible (Figure 1). In short, 382 women were included in the cohort used to describe rates of non-persistence to AI therapy.

The 382 women included in the cohort were followed for a median period of 3.2 years (IQR = [2.6–3.9]). Among these women, 3 had a BC recurrence, 4 developed a new cancer and 8 died during follow-up.

### Non -persistence to AI defined from the pharmacy refill database

**Figure 2:**
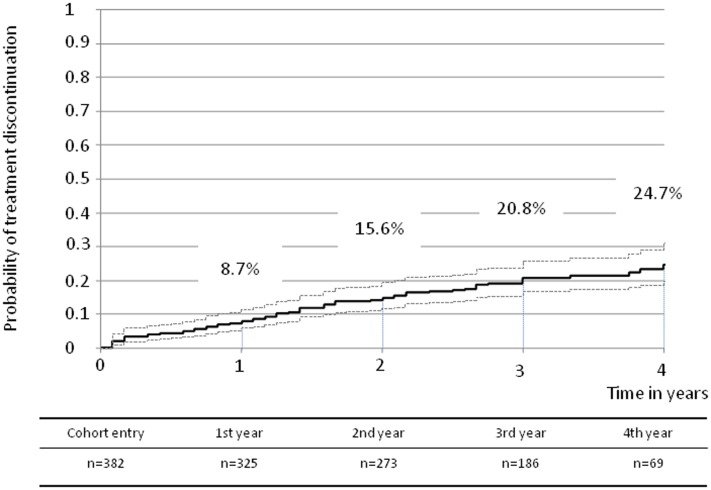
Curves corresponding to cumulative probability of aromatase inhibitor discontinuation with 95% confidence interval (dotted lines) – medication gaps longer than 3 months – ELIPPSE 65 cohort – n = 382.

In addition to the 382 women who filled at least one prescription of AI medication, 57 women (13%) included in the ELIPPSE 65 cohort had positive hormonal receptors but did not fill any prescription. As these women did not receive any supply of AI treatment for BC, they were not eligible for the current analysis. Overall, non-persistence went from 8.7% (95%CI: 6.2–12.1) at 1 year, to 15.6% (95%CI: 12.2–19.8) at 2 years, 20.8% (95%CI: 16.7–25.6) at 3 years, and 24.7% (95%CI: 19.5–31.0) at 4 years (Figure 2).

In particular, 35 of the 382 women switched from AIs to tamoxifen. Among continuous users of AI treatment, 93.5% (n = 357) had more than 80% of days covered (Table 1).

**Table 1:** Treatment coverage according to duration of treatment in the ELIPPSE 65 cohort – n = 382.

Time period	Entire follow-up	1^st^ year	2^nd^ year*	3^rd^ year**	4^th^ year***
n	382	382	310	256	162
Mean MPR$ (SD)	95.0 (8.6)	96.8 (8.3)	96.9 (6.5)	96.6 (5.8)	97.2 (4.4)
> = 80% of days covered n(%)	357 (93.5)	365 (95.5)	300 (96.8)	249 (97.3)	161 (99.4)

Medication Possession Ratio.

Calculated for women with more than one year of treatment.

Calculated for women with more than two years of treatment.

Calculated for women with more than three years of treatment.

### Determinants of non-persistence to AI

To study determinants of non-persistence to AI therapy, we excluded another 33 women who had no data collected in first face-to-face interview and 18 who discontinued their treatment or were censored before first face-to-face interview (Figure 1). We conducted the analysis after restricting our sample to women who reported they were taking their medication without outside help. Determinants of treatment discontinuation were thus studied for 292 women. Furthermore, CAM was found to be used only by women between 65 to 75 years old. Thus, the multivariate analysis was conducted among the 233 women whose age was lower than 75.

Patients' characteristics are described in Table 2 for this sample of 233 women. At the time of diagnosis, women's mean age was 71.8 (sd: 4.2). Stage 1 of BC was diagnosed in 69.1% of women (n = 161), stage 2 in 24.0% (n = 56) and stage 3 in 5.2% (n = 12). With respect to geriatric assessment, most women did not present any cognitive impairment as defined by the mini-COG questionnaire. Comorbidities were reported by 50.6% of women (n = 118). More than 3 different types of medication were received by almost half of the women (49.8%). A mere 13.7% of women reported having been involved in the decision to take hormonal therapy.

**Table 2:** Population characteristics and factors associated with aromatase-inhibitors discontinuation in the ELIPPSE 65 cohort of women aged 74 or less – n = 233.

	n (%)	Crude HR*	p-value
		[95% CI]	
***Socio-demographic characteristics at diagnosis***			
Level of education			
Less than high school certificate	154 (66.1)	1	0.39
High school certificate or higher	77 (33.0)	1.38 [0.66–2.87]	
Missing value	2 (0.9)	-	
Former occupation			0.65
Farmer, craftswoman, or business owner	12 (5.1)	1	
Executive manager	88 (37.8)	0.46 [0.12–1.66]	
Manual worker or employee	104 (44.6)	0.46 [0.13–1.62]	
Housewife	23 (9.9)	0.67 [0.15–2.99]	
Unknown	6 (2.6)	-	
***Medical and treatment-related characteristics***			
PTNM stage			0.95
I	161 (69.1)	1	
II	56 (24.0)	1.11 [0.47–2.61]	
III	12 (5.2)	0.84 [0.11–6.32]	
Missing value	4 (1.7)	-	
Breast surgery			
Breast conserving surgery	195 (85.5)	1	
Mastectomy	33 (14.5)	1.03 [0.36–2.95]	0.96
Chemotherapy			
No	169 (72.5)	1	
Yes	64 (27.5)	0.64 [0.24–1.69]	0.35
Radiotherapy			
No	7 (3.0)	1	
Yes	226 (97.0)	0.49 [0.11–2.07]	0.38
Self-reported co-morbidities			
No	115 (49.4)	1	
Yes	118 (50.6)	1.59 [0.76–3.35]	0.21
Polypharmacy (> = 4 types of medications)			
No	117 (50.2)	1	
Yes	116 (49.8)	0.48 [0.22–0.98]	0.05
Use of complementary and alternative medicine for breast cancer care			
No	196 (84.1)	1	
Yes	37 (15.9)	3.11 [1.45–6.65]	0.01
***Psychosocial characteristics***			
Clear and sufficient information about disease provided at diagnosis			
No	7 (3.0)	-	
Yes	226 (97.0)	-	-
Involvement in the decision to take hormonal therapy			
No	201 (86.3)	1	
Yes	32 (13.7)	0.98 [0.34–2.80]	0.96
***Geriatric assessment at 10 months***			
ADL disability			
Yes	20 (8.6)	1	
No	213 (91.4)	1.06 [0.32–3.46]	0.93
IADL disability			
Yes	46 (19.7)	1	
No	187 (80.3)	0.61 [0.21–1.75]	0.33
Cognitive impairment (Mini-COG)			
Yes	34 (14.6)	1	
No	196 (84.1)	0.77 [0.29–2.01]	0.60
Unknown	3 (1.3)	-	
Mild or severe depressive symptoms (GDS-15)			
No	204(87.5)	1	
Yes	26 (11.2)	1.30 [0.45–3.73]	0.64
Unknown	3 (1.3)	-	

Hazard Ratio.

In the univariate analysis, we found that cancer characteristics were not associated with persistence to treatment. Polypharmacy decreased the risk of AI therapy discontinuation (Hazard Ratio, HR = 0.5; 95%CI: 0.2–1.0). Conversely, the use of CAM for BC treatment increased the risk of treatment discontinuation (HR = 3.1; 95%CI: 1.5–6.7).

In the multivariate analysis (Table 3), CAM use (HR = 3.2; 95%CI: 1.5–6.9), presence of comorbidities (HR = 2.2; 95%CI: 1.0–4.8) and absence of polypharmacy (HR = 0.4; 95%CI: 0.2–0.9) were independent predictors of treatment discontinuation.

**Table 3:** Independent factors associated with aromatase inhibitor intake discontinuation women aged 74 or less – n = 233.

	n (%)	Adjusted HR*	p-value
		(95% CI)	
Complementary and alternative medicine for breast cancer care			
No	196 (84.1)	1	
Yes	37 (15.9)	3.20 [1.49–6.86]	0.00
Self-reported co-morbidities			
No	115 (49.4)	1	
Yes	118 (50.6)	2.22 [1.03–4.78]	0.04
Polypharmacy (> = 4 types of medications)			
No	117 (50.2)	1	
Yes	116 (49.8)	0.40 [0.18–0.88]	0.02

– calculated in a Cox proportional hazard model. Hazard ratio

### Self-reports of non-adherence measured in patient questionnaire

Of the 292 women who were on treatment upon taking the questionnaire, 7 (2.4%) reported that they did not receive any hormonal therapy. When specifying medication names, 29 (9.9%) declared that they did not receive any anastrozole, letrozole or exemestane treatment. This was not found to be associated with treatment discontinuation.

Among the 37 women (10.7%) who reported having forgotten to take their hormonal treatment at least once, the most frequent reason mentioned (29.7%) was not being at home when supposed to take the treatment. Another 9 women (3.0%) declared that they voluntarily interrupted their treatment in the 30 days prior to taking the questionnaire: 2 felt that the treatment was ineffective; 2 declared that they felt better; 2 suffered from side effects; 5 feared suffering from side effects; and 1 did not take the treatment because she was ill that day.

## Discussion

AI treatment was prematurely discontinued by a large proportion of the women under study. Elderly women using complementary or alternative medicine or suffering from comorbidities were more likely to discontinue their treatment, whereas women with polypharmacy were less likely to discontinue.

We have recently conducted a metaregression analysis that synthesizes the available data on adherence to hormonal therapy in BC [15], based on 29 observational studies selected from a comprehensive qualitative review [14]. Taking into account duration and as well as study methodology, we have shown non-persitence to AI increased from 11.7% at 1 year to 31.3 at 5 year. Rates of non-persistence reported in this metaregression match closely treatment discontinuation rates in this study. The wide variability of previously reported rates of treatment discontinuation, ranging from a low of 6% to a high of 40% at one year [13], [26], [27], [28], [29], [30], [31] is mostly due the data source used in the studies. The source of data has indeed been reported to explain over 68% of the variation in measures observed between studies [15].

Studies show that CAM is often used by cancer patients [32]. The prevalence of CAM use has been reported to reach 17% in older patient [33]. Little data is available for France, yet CAM also appears to be widely used in the country [34]. CAM use presents two problems. First, in other diseases such as AIDS, studies have shown that CAM use reduces treatment adherence [35], [36]. Owen-Smith et al. [37] reported that HIV-positive women using CAM are 1.69 times more likely not to adhere to their treatment as compared to non-CAM users. Second, there are possible drug interactions between CAM and cancer therapies. In a previous study on cancer patients in France, Thomas-Schoemann et al. reported that most CAM used corresponded to anti-oxydants that could possibly generate drug interactions [34]. This is a particular concern for older patients, who are at increased risk of drug interactions notably due to polypharmacy. In our study, over 50% of patients received four or more types of medication, which put them at high risk of drug interaction. Physicians should pay attention to these two points, as CAM use is disclosed by patients to MDs in only 20% to 40% of cases [34], [38]. In HIV studies, patients do not disclose this information mostly because they believe it is not relevant, or because physicians do not ask about CAM [38]. Further studies are needed to evaluate more precisely the impact of CAM use on adherence in cancer patients, and to improve patient-physician communication on this topic.

As was previously reported for tamoxifen or other treatments in elderly populations [39], [40], polypharmacy was found to be associated with increased adherence to treatment in our study. Adherence may be higher due to the development of daily routines among patients who have complex prescription regimens. However, Neugut et al. [30] found the opposite association. These conflicting results may be explained by differences in health care systems and medication insurance schemes. In the study by Neugut et al., cost issues associated with AI treatment predicted lower rates of persistence [30]. When patients' insurance schemes do not provide full drug coverage, higher pill burdens lead to higher co-payments. In France, BC patients receive their treatment free of charge; hence polypharmacy was not associated with economic burden for patients in our study.

Our study has several strengths. First, since the French system provides universal health coverage, our study is population-based - i.e. it does not focus on any specific socio-economic group. Second, we had access to several data sources, namely NHIS databases, medical records, and data obtained through face-to-face interviews with patients. This ought to be emphasized because even though most database studies in pharmacoepidemiology use high quality databases, they are rarely able to link these data with medical records or with questionnaires measuring patient-reported outcomes or psychosocial variables. By combining data from different sources, we were able to identify women who were taking their medication without outside help, as well as study the association between CAM use (which is not recorded in pharmacy databases) and a reliable measure of adherence to AI. Among older women, studies on treatment adherence must take into account whether the patient is able to take her medication with or without outside help. Persistence and its determinants are, indeed, likely to differ if treatment is taken without outside help, or if it is provided by a family member or a caretaker.

Two limitations of our study bear mentioning. First, we did not measure real adherence or persistence to medication. Accurate measures of adherence can nonetheless be obtained by using prescription refill rates in a closed pharmacy system such as the French one [22], [41]. Measuring adherence in this way is not equivalent to measuring what the patient actually ingests, but it limits distortions caused by memory bias or by the desire to give socially acceptable answers [42]. Second, we were not able to measure in an unbiased manner the association between side effects and treatment discontinuation. Since side effects were evaluated at 10 months and at 2 years after diagnosis whereas prescription refills were evaluated continuously, the inclusion of a ‘side effects’ variable in our statistical model would have created a protopathic bias. Indeed, because women with less side effects may be women who have previously discontinued their treatment, a spurious association between increased side effects and increased adherence might have appeared in the analysis. The use of a time-dependent model was limited by the lack of variability over time of the ‘side effects’ variable, which was measured only twice at a fixed point in time.

Some elderly patients may be using CAM not as a complementary treatment, but as an alternative to conventional medicine. Health care providers need to be aware of CAM use among their patients, and of its possible impact on adherence to prescribed medication. As only a minority of patients spontaneously disclose CAM use to their physician, an open discussion between health care providers and patients is required to assess CAM use, discuss alternatives and emphasize that CAM should not replace anti-hormonal treatment. The saying “tell your doctor about all your prescription and over-the-counter medications, vitamins, minerals, herbal products, and drugs prescribed by other doctors” should be repeated continuously. Improving patient-physician communication on CAM use may improve hormonal therapy adherence.

## References

[1] Partridge AH, Avorn J, Wang PS, Winer EP (2002) Adherence to therapy with oral antineoplastic agents. J Natl Cancer Inst 94: 652–661. 11983753 10.1093/jnci/94.9.652

[2] Ruddy K, Mayer E, Partridge A (2009) Patient adherence and persistence with oral anticancer treatment. CA Cancer J Clin 59: 56–66. 19147869 10.3322/caac.20004

[3] Early Breast Cancer Trialists' Collaborative Group (2005) Effects of chemotherapy and hormonal therapy for early breast cancer on recurrence and 15-year survival: an overview of the randomised trials. Lancet 365: 1687–1717. 15894097 10.1016/S0140-6736(05)66544-0

[4] Coombes RC, Hall E, Gibson LJ, Paridaens R, Jassem J, et al (2004) A randomized trial of exemestane after two to three years of tamoxifen therapy in postmenopausal women with primary breast cancer. N Engl J Med 350: 1081–1092. 15014181 10.1056/NEJMoa040331

[5] Forbes JF, Cuzick J, Buzdar A, Howell A, Tobias JS, et al (2008) Effect of anastrozole and tamoxifen as adjuvant treatment for early-stage breast cancer: 100-month analysis of the ATAC trial. Lancet Oncol 9: 45–53. 18083636 10.1016/S1470-2045(07)70385-6

[6] Goss PE, Ingle JN, Martino S, Robert NJ, Muss HB, et al (2003) A randomized trial of letrozole in postmenopausal women after five years of tamoxifen therapy for early-stage breast cancer. N Engl J Med 349: 1793–1802. 14551341 10.1056/NEJMoa032312

[7] Howell A, Cuzick J, Baum M, Buzdar A, Dowsett M, et al (2005) Results of the ATAC (Arimidex, Tamoxifen, Alone or in Combination) trial after completion of 5 years' adjuvant treatment for breast cancer. Lancet 365: 60–62. 15639680 10.1016/S0140-6736(04)17666-6

[8] Crivellari D, Sun Z, Coates AS, Price KN, Thurlimann B, et al (2008) Letrozole compared with tamoxifen for elderly patients with endocrine-responsive early breast cancer: the BIG 1-98 trial. J Clin Oncol 26: 1972–1979. 18332471 10.1200/JCO.2007.14.0459

[9] Burstein HJ, Prestrud AA, Seidenfeld J, Anderson H, Buchholz TA, et al (2010) American Society of Clinical Oncology clinical practice guideline: update on adjuvant endocrine therapy for women with hormone receptor-positive breast cancer. J Clin Oncol 28: 3784–3796. 20625130 10.1200/JCO.2009.26.3756 PMC5569672

[10] Goldhirsch A, Ingle JN, Gelber RD, Coates AS, Thurlimann B, et al (2009) Thresholds for therapies: highlights of the St Gallen International Expert Consensus on the primary therapy of early breast cancer 2009. Ann Oncol 20: 1319–1329. 19535820 10.1093/annonc/mdp322 PMC2720818

[11] Dezentje V, Van Blijderveen NJ, Gelderblom H, Putter H, Van Herk - Sukel MP, et al (2009) Concomitant CYP2D6 inhibitor use and tamoxifen adherence in early-stage breast cancer: A pharmacoepidemiologic study. J Clin Oncol 27: 18s. 10.1200/JCO.2009.25.0894 20385997

[12] McCowan C, Shearer J, Donnan PT, Dewar JA, Crilly M, et al (2008) Cohort study examining tamoxifen adherence and its relationship to mortality in women with breast cancer. Br J Cancer 99: 1763–1768. 18985046 10.1038/sj.bjc.6604758 PMC2600703

[13] Hershman DL, Shao T, Kushi LH, Buono D, Tsai WY, et al (2011) Early discontinuation and non-adherence to adjuvant hormonal therapy are associated with increased mortality in women with breast cancer. Breast Cancer Res Treat 126: 529–537. 20803066 10.1007/s10549-010-1132-4 PMC3462663

[14] Murphy CC, Bartholomew LK, Carpentier MY, Bluethmann SM, Vernon SW (2012) Adherence to adjuvant hormonal therapy among breast cancer survivors in clinical practice: a systematic review. Breast Cancer Res Treat 134: 459–478. 22689091 10.1007/s10549-012-2114-5 PMC3607286

[15] Huiart L, Ferdynus C, Giorgi R (2013) A meta-regression analysis of the available data on adherence to adjuvant hormonal therapy in breast cancer: summarizing the data for clinicians. Breast Cancer Res Treat 138: 325–328. 23400580 10.1007/s10549-013-2422-4

[16] Huiart L, Bouhnik AD, Rey D, Tarpin C, Cluze C, et al (2012) Early discontinuation of tamoxifen intake in younger women with breast cancer: Is it time to rethink the way it is prescribed? Eur J Cancer 48: 1939–1946. 22464016 10.1016/j.ejca.2012.03.004

[17] Martin-Latry K, Begaud B (2010) Pharmacoepidemiological research using French reimbursement databases: yes we can!. Pharmacoepidemiology and drug safety 19: 256–265. 20128015 10.1002/pds.1912

[18] Katz S (1983) Assessing self-maintenance: activities of daily living, mobility, and instrumental activities of daily living. J Am Geriatr Soc 31: 721–727. 6418786 10.1111/j.1532-5415.1983.tb03391.x

[19] Fillenbaum GG, Smyer MA (1981) The development, validity, and reliability of the OARS multidimensional functional assessment questionnaire. J Gerontol 36: 428–434. 7252074 10.1093/geronj/36.4.428

[20] Borson S, Scanlan J, Brush M, Vitaliano P, Dokmak A (2000) The mini-cog: a cognitive ‘vital signs’ measure for dementia screening in multi-lingual elderly. Int J Geriatr Psychiatry 15: 1021–1027. 11113982 10.1002/1099-1166(200011)15:11<1021::aid-gps234>3.0.co;2-6

[21] Almeida OP, Almeida SA (1999) Short versions of the geriatric depression scale: a study of their validity for the diagnosis of a major depressive episode according to ICD-10 and DSM-IV. Int J Geriatr Psychiatry 14: 858–865. 10521885 10.1002/(sici)1099-1166(199910)14:10<858::aid-gps35>3.0.co;2-8

[22] Andrade SE, Kahler KH, Frech F, Chan KA (2006) Methods for evaluation of medication adherence and persistence using automated databases. Pharmacoepidemiol Drug Saf 15: 565–574 discussion 575-567. 16514590 10.1002/pds.1230

[23] Cramer JA, Roy A, Burrell A, Fairchild CJ, Fuldeore MJ, et al (2008) Medication compliance and persistence: terminology and definitions. Value Health 11: 44–47. 18237359 10.1111/j.1524-4733.2007.00213.x

[24] Fisher B, Costantino J, Redmond C, Poisson R, Bowman D, et al (1989) A randomized clinical trial evaluating tamoxifen in the treatment of patients with node-negative breast cancer who have estrogen-receptor-positive tumors. N Engl J Med 320: 479–484. 2644532 10.1056/NEJM198902233200802

[25] Love RR (1990) Prospects for antiestrogen chemoprevention of breast cancer. J Natl Cancer Inst 82: 18–21. 2403437 10.1093/jnci/82.1.18

[26] Partridge AH, LaFountain A, Mayer E, Taylor BS, Winer E, et al (2008) Adherence to initial adjuvant anastrozole therapy among women with early-stage breast cancer. J Clin Oncol 26: 556–562. 18180462 10.1200/JCO.2007.11.5451

[27] Ziller V, Kalder M, Albert US, Holzhauer W, Ziller M, et al (2009) Adherence to adjuvant endocrine therapy in postmenopausal women with breast cancer. Ann Oncol 20: 431–436. 19150950 10.1093/annonc/mdn646

[28] Hershman DL, Kushi LH, Shao T, Buono D, Kershenbaum A, et al (2010) Early discontinuation and nonadherence to adjuvant hormonal therapy in a cohort of 8,769 early-stage breast cancer patients. J Clin Oncol 28: 4120–4128. 20585090 10.1200/JCO.2009.25.9655 PMC2953970

[29] Huiart L, Dell'Aniello S, Suissa S (2011) Use of tamoxifen and aromatase inhibitors in a large population-based cohort of women with breast cancer. Br J Cancer 104: 1558–1563. 21522148 10.1038/bjc.2011.140 PMC3101914

[30] Neugut AI, Subar M, Wilde ET, Stratton S, Brouse CH, et al (2011) Association between prescription co-payment amount and compliance with adjuvant hormonal therapy in women with early-stage breast cancer. J Clin Oncol 29: 2534–2542. 21606426 10.1200/JCO.2010.33.3179 PMC3138633

[31] Nekhlyudov L, Li L, Ross-Degnan D, Wagner AK (2011) Five-year patterns of adjuvant hormonal therapy use, persistence, and adherence among insured women with early-stage breast cancer. Breast Cancer Res Treat 130: 681–689. 21842245 10.1007/s10549-011-1703-z

[32] Richardson MA, Sanders T, Palmer JL, Greisinger A, Singletary SE (2000) Complementary/alternative medicine use in a comprehensive cancer center and the implications for oncology. J Clin Oncol 18: 2505–2514. 10893280 10.1200/JCO.2000.18.13.2505

[33] Maggiore RJ, Gross CP, Togawa K, Tew WP, Mohile SG, et al (2012) Use of complementary medications among older adults with cancer. Cancer 118: 4815–4823. 22359348 10.1002/cncr.27427 PMC3366170

[34] Thomas-Schoemann A, Alexandre J, Mongaret C, Azibi S, Dauphin A, et al (2011) [Use of antioxidant and other complementary medicine by patients treated by antitumor chemotherapy: a prospective study]. Bull Cancer 98: 645–653. 21636352 10.1684/bdc.2011.1375

[35] Jernewall N, Zea MC, Reisen CA, Poppen PJ (2005) Complementary and alternative medicine and adherence to care among HIV-positive Latino gay and bisexual men. AIDS Care 17: 601–609. 16036246 10.1080/09540120512331314295

[36] Knippels HM, Weiss JJ (2000) Use of alternative medicine in a sample of HIV-positive gay men: an exploratory study of prevalence and user characteristics. AIDS Care 12: 435–446. 11091776 10.1080/09540120050123837

[37] Owen-Smith A, Diclemente R, Wingood G (2007) Complementary and alternative medicine use decreases adherence to HAART in HIV-positive women. AIDS Care 19: 589–593. 17505918 10.1080/09540120701203279

[38] Eisenberg DM, Kessler RC, Van Rompay MI, Kaptchuk TJ, Wilkey SA, et al (2001) Perceptions about complementary therapies relative to conventional therapies among adults who use both: results from a national survey. Ann Intern Med 135: 344–351. 11529698 10.7326/0003-4819-135-5-200109040-00011

[39] Lash TL, Fox MP, Westrup JL, Fink AK, Silliman RA (2006) Adherence to tamoxifen over the five-year course. Breast Cancer Res Treat 99: 215–220. 16541307 10.1007/s10549-006-9193-0

[40] Balkrishnan R (1998) Predictors of medication adherence in the elderly. Clin Ther 20: 764–771. 9737835 10.1016/s0149-2918(98)80139-2

[41] Osterberg L, Blaschke T (2005) Adherence to medication. N Engl J Med 353: 487–497. 16079372 10.1056/NEJMra050100

[42] Choo PW, Rand CS, Inui TS, Lee ML, Cain E, et al (1999) Validation of patient reports, automated pharmacy records, and pill counts with electronic monitoring of adherence to antihypertensive therapy. Medical care 37: 846–857. 10493464 10.1097/00005650-199909000-00002

